# The pattern and socio-cultural determinants of intimate partner violence in a Nigerian rural community

**DOI:** 10.4102/phcfm.v13i1.2435

**Published:** 2021-06-01

**Authors:** Israel C. Ikekwuibe, Collins E.M. Okoror

**Affiliations:** 1 Department of Obstetrics and Gynaecology, University of Benin Teaching Hospital, Benin City, Nigeria

**Keywords:** intimate partner violence, burden, sociocultural determinants, rural community, Nigeria

## Abstract

**Background:**

Intimate partner violence (IPV) refers to a violation of women’s reproductive rights as it impacts on their sexual and reproductive health autonomy.

**Aim:**

In this study, we aimed at assessing the pattern of IPV and the socio-cultural determinants and predictors of partner violence in a rural community setting where the bulk of the abuse prevails.

**Setting:**

This study was conducted in a rural community in Southern Nigeria.

**Methods:**

This study employed a mixed method comprising seven focus group discussions (FGDs) and quantitative components. The cross-sectional survey was conducted amongst 209 ever married or cohabited females in 2018 using the World Health Organization (WHO) multi-country survey questionnaire adapted to the study objectives. Data analysis was conducted by using IBM SPSS v21.0. The level of significance was set at *p* < 0.05.

**Results:**

The overall IPV prevalence was 79.4%. The prevalence of partner’s controlling behaviour, emotional IPV, physical IPV and sexual IPV was 62.6%, 55.98%, 49.3% and 2.6%, respectively. Membership of an interest group was protective against IPV (OR = 0.430, 95% CI = 0.193–0.957) whilst the belief that a good wife obeys her partner even if she disagrees (OR = 9.201, 95% CI = 1.299–65.194) and the belief that it is the wife’s obligation to have sex with the husband even if she doesn’t feel like (OR = 2.356, 95% CI = 1.049–5.288) were risk factors.

**Conclusion:**

The burden of IPV in the studied rural community is enormous. There should be public enlightenment to desensitise people regarding the erroneous views that encourage partner violence. We encourage women to become a part of social groups that can enhance their education and empowerment.

## Introduction

As clearly defined by the World Health Organisation (WHO), intimate partner violence (IPV) refers to any behaviour within an intimate relationship that causes physical, psychological or sexual harm to those in the relationship.^1^ It is one of the most common human rights violations and epitomises, in a very vivid manner, the inequality between men and women. The ever-increasing quest for power, autonomy, patriarchal tendencies and economic insecurities accentuates the practice.^2,3^ It is currently becoming a major public health problem in most countries, but evidence from representative, community-based studies is said to be limited.^4^

Intimate partner violence undermines the physical, mental, social, sexual and reproductive well-being of women.

The prevalence of IPV varies depending on the setting of the survey. The prevalence varies between rural and urban, community- and hospital-based studies and developing and developed countries. A growing body of evidence highlights an increase in the prevalence of IPV in developing countries.^5,6,7^ In sub-Saharan Africa, the burden of IPV has been quoted as between 20% and 71%.^4,8,9^

Intimate partner violence assumes a variety of forms including physical assaults such as hits, kicks, beating, throwing of objects or corrosive substance; psychological assaults like intimidation, belittling, humiliation, threats, restricting contact with her family of birth, suspicion of infidelity and sexual assaults like forced intercourse and degrading or humiliating sexual acts.^10,11^

Personal and partner factors, partnership status and cultural factors, all interact to either protect or increase the risk of IPV. Studies have enumerated factors like being young, low socio-economic status, unmarried status, history of violence amongst parents of the partner, women contributing a greater share to the family income, drug and alcohol abuse by the partner, dysfunctional and unhealthy relationships characterised by inequality, power imbalance and conflict as some of the factors responsible for IPV.^11,12,13,14,15^ Studies have also shown that victims justify partner’s violence under certain circumstances. This is generally because of a huge deal of traditional and societal acceptance of partner violence even from the abused. In an African study by Koenig et al.^4^ in a rural area in South-western Uganda, it is intriguing to observe that for almost all behaviours cited by the respondents (males and females) as justifiable for physical violence, a greater proportion of women justified them than did the men. These behaviours included refusal to have sex with her partner, contraception without permission from her partner and infidelity by the female partner. In a research conducted by Owoaje and OlaOlorun^16^ it was observed that respondents with attitudes that justify IPV were at risk of experiencing greater physical violence. However, the underpinning socio-cultural determinants of IPV are yet to be extensively studied.

Research studies on IPV are mainly health facility-based in which a small fraction of victims present basically because of the sustained injury and for fear of being stigmatised in the majority; thus, this does not mirror what is obtained in the community. Another drawback of such a facility-based study is selection bias and non-randomisation. Clearly, gaining a full understanding of the socio-cultural factors that propel IPV may not be achieved in a health facility-based study as the victims seldom report, partly because of the prevailing cultural perceptions and the probable judicial sanctioning of the intimate partner. Few community-based studies that have addressed IPV in the past were in urban settings.^17^ It is no wonder why IPV still persists even in growing proportions despite efforts being made to curb it. As most of the socio-cultural factors associated with IPV are common in the rural communities, a rural community-based research is imperative if any meaningful progress has to be made in addressing the burden of IPV. The objective of this study, therefore, seeks to determine the pattern and socio-cultural determinants of IPV in a rural community in Nigeria.

## Methods

This cross-sectional analytical study conducted in 2018 was a mixed method comprising qualitative (focus group discussions [FGDs]) and quantitative components. It was conducted amongst households in the Ekiadolor community, which is one of the rural communities in the Ovia North East Local Government Area (LGA) of Edo State, Southern Nigeria. Ekiadolor is a community with a total population of about 6100 inhabitants. It is largely rural but possesses some urban area components like tarred roads and public electricity in some parts of the community with a state government-owned district hospital. The society is traditionally patriarchal with domestic violence also being common.^18^ The community youth group merely allows females, whilst the community elders’ group invariably excludes females. This pictorially illustrates what is obtained in most rural communities in developing countries, including Nigeria.

More than 90% of the married female residents in the community are illiterate, with little or no formal education. Different ethnic groups are residents of Ekiadolor, including Bini, Esan, Etsako, Igbo, Urhobo, Ijaw, Isoko and Yoruba, but the Binis constitute about 70% of the population. Despite the various ethnic groups in the community, the local English language (Pidgin English) is widely spoken by the residents and this was used for the interviews. A large part of the working population comprises peasant farmers and petty traders with a crude estimate of income per capita of about $1.00 per day.^18^

A total of seven FGDs (four for males aged 18 and above; and three for ever married/cohabited females between the age of 15 and 49 years) were conducted with participants purposively selected amongst residents of the community. Each FGD consisted of between four and 12 participants. The findings from the FGDs were collated and woven into the WHO multi-country study questionnaire in order to reflect local and cultural peculiarities of IPV. The adapted questionnaire was then used for the quantitative survey.

The sample size determination for the quantitative study was calculated using the formula for prevalence study. A prevalence of 15% from a community-based WHO multi-country study^9^ was used giving a sample size of 196. Considering a 10% attrition rate, a sample size of 220 was arrived at. However, a total of 209 respondents from the proposed 220 participated in the study, giving a response rate of 95%. The participants in this study were 209 ever married/cohabited females and residents of the Ekiadolor community for at least 6 months. Only those who gave consent were recruited for the study. Those who were sick or unable to grant an interview were excluded. A pre-testing of the questionnaire was conducted using 10% of the total sample size and the questionnaire was corrected as required.

A systematic sampling method was used to select the female respondents for the survey. The community is naturally divided into four quadrants by major roads. Fifty-five participants were sampled from each of this quadrant. In each quadrant, every third house was sampled until 55 consenting participants were consecutively recruited. Where there was no eligible person in a house, the next house is moved to.

Quantitative data collection was done making use of the interviewer-administered questionnaire which was converted to computer-assisted personal interviewing (CAPI) software using hand-held mobile devices. Interviewers were trained on the objectives of the study and use of the questionnaire to collect data. Data collection was done between February and August 2018.

All data were computed and analysed by using IBM SPSS for Windows, version 21.0 (Armonk, NY: IBM Corp). Descriptive statistics was used to determine the burden of IPV and bivariate analysis for associations between variables. All the factors that were significantly associated with IPV were run in a multivariate regression model to determine the independent predictors of IPV. The level of statistical significance was set at *p* < 0.05.

### Ethical considerations

The ethical approval for this study was obtained from the Research and Ethics Committee of the College of Medical Sciences, University of Benin, Benin City, Nigeria with protocol number CMS/REC/2017/021. Verbal informed consent was obtained from all the participants after explaining the objectives and procedures of the study to them. The medium of communication was Pidgin English. The participants were assured of confidentiality and privacy during the interview and in data management. The participants were also informed that they had the right to decline participation or withdraw from the study at any given point of time.

## Results

Table 1 shows the socio-demographic profile of the women interviewed. Approximately two-fifth of them have lived in the community for over 5 years. The mean age of the respondents was 32.4 years. A majority (142; 67.9%) had at least a secondary level of education whilst seven (3.3%) of the respondents were without any form of education. Whilst nearly one-fifth of them were not married at the time of the survey, majority of the respondents 142 (67.8%) were married whilst a few 27 (12.3%) were just co-habiting. A greater proportion of them (84; 40.2%) never had any form of marriage ceremony, whereas 77 (33.5%) had only customary marriage.

**TABLE 1:** Socio-demographic characteristics of the respondents.

Variable	Category	Frequency	Percentage
**Number of years in community (*****n*** **= 209)**			
Range (years): 6–25	≤ 5	126	60.3
Mean: 8.38 ± 11.21	> 5	83	39.7
**Age of respondent (years) (*****n*** **= 209)**			
Range: 15–70	< 20	18	8.6
	20–29	74	35.4
	30–39	66	31.6
	40–49	44	21.1
Mean: 32.42 ± 9.49	≥ 50	7	3.4
**Highest educational level (*****n*** **= 209)**	No education	7	3.3
	Primary	60	28.7
	Secondary	100	47.8
	College/tertiary	42	20.1
**Attend group meeting (*****n*** **= 209)**	Yes	107	51.2
	No	102	48.8
**Kind of group (*****n*** **= 107)**	Civic/political	6	5.6
	Social/charitable	9	8.6
	Economic/saving club	15	14.0
	Women organization	25	23.3
	Religious organization	50	46.7
	Others	2	1.8
**Ever been married (*****n*** **= 209)**	Yes	187	89.5
	No	22	10.5
**How long have you been married? (*****n*** **= 187)**			
Range from 1 to 38 years	≤ 5	69	36.9
	6–10	41	21.9
	11–15	22	11.7
Mean: 10.11	> 15	55	29.5
**Current marital status (*****n*** **= 209)**	Married	142	67.9
	Living with a man, not married	27	12.3
	Not married/not living with a man	40	19.1
**Type of marriage ceremony (*****n*** **= 209)**	None	84	40.2
	Civil marriage	26	12.4
	Religious marriage	29	13.9
	Customary marriage	70	33.5

Table 2 shows the incidences of IPV experienced by the respondents. The most experienced controlling IPV as reported by the respondents was their partners insisting on knowing where they were always (69; 33.0%), while their partner ignoring or treating them differently was the least reported (15; 7.1%). Only 78 (37.3%) of them never experienced any form of controlling behaviour. Of the different types of emotional IPV, 110 (52.6%) of the respondents said that they were insulted or made to feel bad about themselves whilst 65 (31.1%) of the respondents were belittled or humiliated in the presence of other people or threatened of being hurt. Sixty-three (30.1%) of them have had all these forms of emotional IPV which have been discussed. Ninety (43.1%) respondents have been slapped or have had hurtful objects hurled at by their partners whilst one (0.5%) of the respondents said that she was choked on purpose. Forty-one (19.6%) were physically assaulted by partners whilst they were pregnant. Four (1.9%) of the respondents have been forced to have sexual intercourse when not willing and two (1.0%) were forced to get involved in degrading or humiliating sexual acts.

**TABLE 2:** Experience and practice of intimate partner violence.

Variable	Category	Frequency	Percent
How often do you quarrel with your husband/partner	Often	23	11.0
	Sometimes	105	50.2
	Rarely	47	22.5
	None	34	16.3
Has your current partner: (Controlling IPV)	Insist on knowing where you are always	69	33.0
	Expect you to ask permission before seeking healthcare	57	27.3
	Prevents you from visiting your friend	47	22.5
	Get angry if you talk to another man	36	17.2
	Is often suspicious that you are unfaithful	22	10.5
	Tries to restrict you from contacting family members	18	8.6
	Ignore you or treat you differently	15	7.1
	None of the above	78	37.3
Has your current husband ever done the following things to you? (Emotional IPV)	Insult you or make you feel bad about yourself	110	52.6
	Do things to scare or intimidate you on purpose	66	31.6
	Belittle or humiliate you in the presences of other people	65	31.1
	Threaten to hurt you or someone you love	65	31.1
	Others	2	1.0
	None of the above	90	43.1
Which of these has your husband/partner ever done before? (Physical IPV)	Slapped you or throw something at you that could hurt you.	90	43.1
	Pushed or shoved you.	56	26.8
	Hit you with his fits or with something that could hurt you.	46	22.0
	Kicked you, dragged you or beaten you up.	8	3.8
	Threaten to use or used a knife, gun or other weapon against you.	3	1.4
	Choked you or burnt you on purpose.	1	0.5
	None of the above	106	50.7
Which of these has your husband/partner ever done before? (Sexual IPV)	Physically forced you to have sexual intercourse when you do not want to	4	1.9
	Forced you to do something sexual that you feel degrading or humiliating	2	1.0
	None of the above	203	97.1
Was there ever a time you were beaten or physically assaulted by your husband/partner when you were pregnant?	Yes	41	19.6
	No	168	80.4

IPV, intimate partner violence.

**FIGURE 1:**
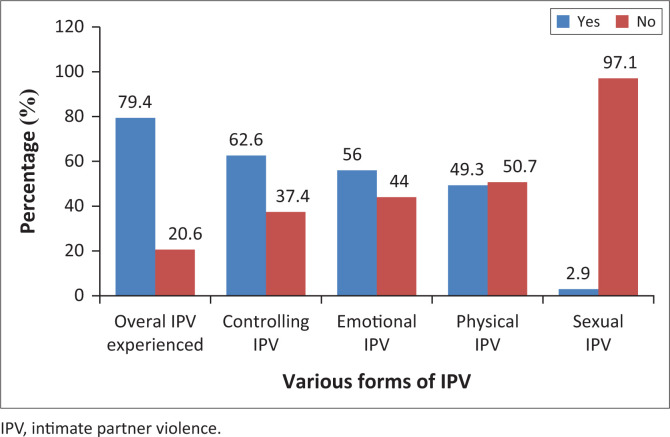
Experience of various forms of intimate partner violence by respondents.

Out of the 209 women interviewed, 166 experienced any one type of IPV. This gives the overall IPV prevalence of 79.4% amongst the population studied. The prevalence of the various forms of IPV amongst the respondents was 62.6%, 56%, 49.3% and 2.9% for partner’s controlling behaviour, emotional IPV, physical IPV and sexual IPV, respectively (Figure 1).

Belonging to a group/association showed a significant association with IPV experience (*p* = 0.017). Also, formalising a marriage had a statistically significant relationship with experience of IPV (*p* = 0.030) (Table 3).

**TABLE 3:** Relationship between respondents’ social demographic characteristics and the experience of intimate partner violence.

Variables	Experienced IPV				*X*^2^	*p*
	Yes		No			
	*n*	%	*n*	%
**Age (in years)**						
< 20	16	84.2	3	15.8	3.393	0.494
20–29	54	73.0	20	27.0		
30–39	55	84.6	10	15.4		
40–49	35	79.5	9	20.5		
≥ 50	6	85.7	1	14.3		
**Educational level achieved**						
No education	6	85.7	1	14.3	2.231	0.526
Primary	48	80.0	12	20.0		
Secondary	82	82.0	18	18.0		
College/tertiary	30	71.4	12	28.6		
**Belong to group/association**						
Yes	78	72.9	29	27.1	5.719	0.017
No	88	86.3	14	13.7		
**Current marital status**						
Married	113	79.6	29	20.4	0.055	0.973
Cohabiting	21	77.8	6	22.2		
Alone	32	80.0	8	20.0		
**Length of marriage (years)**						
£5	72	79.1	19	20.9	4.214	0.746
6–10	30	73.2	11	26.8		
11–15	20	90.9	2	9.1		
16–20	20	76.9	6	23.1		
21–25	9	81.8	2	18.2		
> 25	15	83.3	3	16.7		
**Formal marriage ceremony**						
Civil	25	96.2	1	3.8	8.928	0.030
Religious	19	65.5	10	34.5		
Customary	58	82.9	12	17.1		
None	64	76.2	20	23.8		

IPV, intimate partner violence.

From Table 4, partner’s use of hard drugs and having illicit relationship with another woman had a statistically significant relationship with the experience of IPV (*p* = 0.030 and 0.032, respectively).

**TABLE 4:** Relationship between respondent’s partner socio-demographics and the experience of intimate partner violence.

Variables	*n*	%	Experienced IPV				*X*^2^	*p*
			Yes		No			
			*n*	%	*n*	%
**Age (years)**							
20–29	31	14.8	22	71.0	9	29.0	8.774	0.118
30–39	68	32.5	56	82.4	12	17.6		
40–49	49	23.4	43	87.8	6	12.2		
50–59	26	12.4	22	84.6	4	15.4		
≥60	15	7.2	9	60.0	6	40.0		
Don’t know	20	9.6	14	70.0	6	30.0		
**Level of education**							
No education	2	1.0	1	50.0	1	50.0	4.214	0.378
Primary	26	12.4	21	80.8	5	19.2		
Secondary	96	45.9	81	84.4	15	15.6		
Tertiary	61	29.2	46	75.4	15	24.6		
Don’t know	24	11.5	17	70.8	7	29.2		
**Profession**							
Professional	20	9.6	16	80.0	4	20.0	9.717	0.137
Skilled	58	27.8	45	77.6	13	22.4		
Unskilled	96	45.9	82	85.4	14	14.6		
Military/police	11	5.3	8	72.7	3	27.3		
Looking for job/unemployed	3	1.4	3	100.0	0	0.0		
Retired	9	4.3	5	55.6	4	44.4		
Student	12	5.7	7	58.3	5	41.7		
**Use of alcohol**							
Every day or nearly every day	31	14.8	26	83.9	5	16.1	2.474	0.649
Once or twice a week	23	11.0	18	78.3	5	21.7		
Few times a month	42	20.1	36	85.7	6	14.3		
Never	97	46.4	73	75.3	24	24.7		
Don’t know	16	7.7	13	81.3	3	18.8		
**Use of hard drugs**							
Every day or nearly every day	10	4.8	10	100.0	0	0.0	8.925	0.030
Once or twice a week	4	1.9	4	100.0	0	0.0		
Few times a month	10	4.8	5	50.0	5	50.0		
Never	185	88.5	147	79.5	38	20.5		
**Relationship with another woman (extramarital affair)**							
Yes	56	26.8	47	83.9	9	16.1	6.914	0.032
No	105	50.2	76	72.4	29	27.6		
Maybe	48	23.0	43	89.6	5	10.4		

IPV, intimate partner violence.

Table 5 illustrates the relationship between the participants’ perception and experience of IPV. There was a significant relationship with a belief ‘that a good wife must obey her partner’ and IPV experience (*p* = 0.031). There was also a strong association between the belief in a wife’s unconditional obligation to sex and IPV experience (*p* = 0.002).

**TABLE 5:** Relationship between respondents’ attitude and the experience of intimate partner violence.

Variables	*n*	%	Experienced IPV				*X*^2^	*p*
			Yes		No			
			*n*	%	*n*	%
**A good wife obeys her husband or partner even if she disagrees**								
Agree	169	80.9	139	82.2	30	17.8	6.950	0.031
Disagree	35	16.7	25	71.4	10	28.6		
Don’t know	5	2.4	2	1.2	3	60.0		
**It is important for a man to show wife or partner who is the boss**								
Agree	140	67.0	112	80.0	28	20.0	1.186	0.553
Disagree	64	30.6	51	79.7	13	20.3		
Don’t know	5	2.4	3	60.0	2	40.0		
**A woman should be able to choose her own friends if even her husband disapproves**								
Agree	87	41.6	65	74.7	22	25.3	2.180	0.336
Disagree	188	56.5	98	83.1	20	16.9		
Don’t know	4	1.9	3	75.0	1	25.0		
**It is the wife’ s obligation to have sex with her husband even if she doesn’t feel like it**								
Agree	99	47.3	86	86.9	13	13.1	12.970	0.002
Disagree	108	51.7	80	74.1	28	25.9		
Don’t know	2	1.0	0	0.0	2	100.0		

IPV, intimate partner violence.

Women who belong to any social group were less likely to experience IPV compared to those who do not belong to any (odds ratio [OR] = 0.430, 95% confidence interval [CI] = 0.193–0.957). Respondents who agreed that a good wife must obey her husband/partner even if she disagrees were about nine times more likely to experience IPV compared to those who said that they do not know (OR = 9.201, 95% CI = 1.299–65.194). The women who agreed to the fact that it is the wife’s obligation to have sexual relations with her partner even if she does not feel like, were over twice more likely to experience IPV compared to those who disagree/do not know (OR = 2.356, 95% CI = 1.049–5.288) (Table 6).

**TABLE 6:** Predictors of women experiencing intimate partner violence.

Variables	B	OR	95% CI	*p*
**Belong to group/association**				
Yes	−0.845	0.430	0.193–0.957	0.039
No	-	1	-	-
**Relationship with another woman**				
Yes	−0.258	0.773	0.222–2.695	0.686
No	−1.081	0.339	0.114–1.014	0.053
**Formal marriage ceremony**				
Civil	−0.366	0.694	0.284–1.692	0.421
Religious	1.496	4.464	0.527–37.837	0.170
Customary	−0.696	0.499	0.173–1.441	0.199
None	-	1	-	-
**A good wife obeys her husband/partner even if she disagrees**				
Agree	2.219	9.201	1.299–65.194	0.026
Disagree	1.888	6.608	0.818–53.402	0.077
Don’t know	-	1	-	-
**It is the wife’s obligation to have sex with her husband even if she doesn’t feel like it**				
Agree	0.857	2.356	1.049–5.288	0.038
Disagree/don’t know	-	1	-	-

OR, odds ratio; CI, confidence interval.

## Discussion

Every cultural system has its belief related to the importance of relationship/home, and the succour and security it affords. Yet for a proportion of women, the home is a place of untold torture and intimidation. Because majority of the violence takes place behind closed doors and to many, it is a phenomenon not meant for public discourse, there may lie an underpinning socio-cultural dimension to it. This is the premise on which this study was conceptualised and conducted.

The prevalence of IPV amongst the ever-partnered women in this study was 79.4%. This figure is comparable to a lifetime prevalence of partner violence reported in several regions in Nigeria.^8,17,19^ The prevalence of 79.4% is higher in comparison to a prevalence of 53.3% obtained earlier in a study in Benin City, Nigeria^20^ despite similar measures applied in both studies. This difference may be accounted for by the population sampled. Whilst this earlier study was conducted amongst antenatal clinic attendees, the index study was a community-based study drawing its strength from the heterogenicity of the population and rural community where IPV is more likely to be prevalent.

The prevalence of physical IPV was 49.3%. The implication is that nearly one out of every two women of reproductive age in the community experienced one form of physical violence or the other. This is similar to that reported in Ethiopia province, Peru City and United Republic of Tanzania province, with physical IPV prevalence of 48.7%, 48.6% and 46.7%, respectively.^9^ It is noteworthy that the only two African countries (Ethiopia and Tanzania) represented in this WHO multi-country study ranked highest in the prevalence of physical IPV amongst the other countries studied. These reports go to show that IPV is widely condoned in many African settings where it is believed that it is acceptable for the husband to chastise his wife for whatever reason is deeply embedded in the culture.^4,14,21,22^

The observed prevalence of emotional/psychological IPV of 55.9% is higher than that obtained in various local studies.^4,17,20,23^ This high figure may be reflective of the general prevalence of IPV in the understudied community because there is often an overlap between partner violence categories.

The prevalence of 62.2% of controlling attitude exhibited by the male partners is alarmingly high. This may be attributable to the highly traditionally patriarchal nature of the Ekiadolor community as noted in a previous study^18^ in which the male folks try to restrict the range of social freedom granted to their female folks. Moreover, this high prevalence of controlling attitude reflects the magnitude of other types of violence against women in any community as has been shown that men who physically abuse their partners also exhibit greater tendencies of controlling behaviour than men who do not.^9,10^

The prevalence of sexual IPV in the index study of 2.9% greatly contrasts that from other African studies which quoted much higher figures.^20,23,24,25^ These wide disparities may be as a result of differences in defining what constitutes sexual violence from an intimate partner. Moreover, some cultures abhor discussing sexual relations between intimate partners. Therefore, cases of sexual violence are not often reported, and this may be responsible for the recorded low prevalence.

Whilst formal education is being conferred by formal institutions, there may be some forms of organisations/institutions where informal learning is imparted. Different organisations focus on different interests, for example, political groups, social clubs and women empowerment groups, and majority of the women in this study (51.2%) belonged to one of such groups in the community. Contrary to the previous report that female’s level of empowerment and social support contributes to the risk of IPV,^8,19^ belonging to a group in this study was protective against IPV experience. The enlightenment and empowerment derivable from such meetings equipped the woman with measures to overcome or prevent the occurrence of IPV.

Partners’ low educational status, unskilled profession, alcohol/psychoactive substance use, multiple sexual partners, exposure to parental violence etc. are partner-dependent factors known to be associated with domestic violence. This study showed no significant association between partner’s level of education, profession and alcohol use and IPV experience in this study. However, similar to the findings of Owoaje and OlaOlorun^16^ the use of psychoactive drugs had a significant association with IPV. Also, the partner’s illicit relationship with another woman was found to be statistically related with IPV experience. The respondent’s suspicion of the partner’s infidelity attracts some forms of punishment from the partner or a strained relationship resulting from partner violence leads to extramarital affairs. These factors were, however, not independently predictive of IPV.

There was observed significant association between the solemnisation of marriage amongst the respondents and IPV experience. It may be that formalisation of union haven secured the marriage, negatively emboldens the women as they are more likely to challenge their partners’ authority and therefore be prone to partner violence. This, however, did not show independent association with IPV experience.

Belief systems and cultural norms have been found to play important roles in determining the prevalence of IPV. Bearing in mind the fact that one’s perception is a progeny of one’s culture, certain partner violence experienced by some women may be a result of the traditional views of violence they endorse. This study evaluated the role of belief systems in determining the prevalence of IPV. Over four-fifth of the respondents agreed that a good wife must obey her husband even if she disagrees with him. This was predictive of IPV experience as they were more likely to experience IPV. Nearly one-half of the respondents agreed that the wife is obligated to have sex with the partner even if she did not feel like it. These women were twice more likely to experience IPV compared to those who disagree. These findings are consistent with observations in several studies on domestic violence in which the proportion of women agreeing with a particular justification for abuse was higher amongst women who had experienced IPV than those who had not.^4,9,14,22,26^ This may indicate that women either learn to rationalise abuse in circumstances where they themselves are the sufferers, or that women are more prone to abuse in communities where community-level norms subscribe to the acceptability of gender violence.^9^ In fact, studies from South Africa and Russia report the ‘normalization’ of violence and the use of such violence as a means of domestic disharmony resolution.^27,28^ Cultural justifications for violence are often given in many countries, usually following traditional perceptions of the acceptable roles of men and women. It further explains the fact that African culture permits a husband’s ‘ownership’ of his spouse.^19^

The burden of IPV in our community is still very high despite various mitigating measures that are being put in place by government and non-governmental organisations. In all the attitudes/beliefs inquired, more women had a supportive attitude towards IPV, and partner violence was common amongst them. This is consistent with the findings of several workers on partner violence.^4,9,18,29,30^ Studies have consistently shown that overall acceptance that partner-abuse is justified for some reasons significantly increases the experience of violence. This study has given further credence to this discovery. This belief system strongly predicts partner violence. Invariably, these approvals of female abuse illustrate cultural tolerance to gender inequality. Belonging to a group was the only factor that was protective against IPV in this study.

### Strengths and limitations

One of the strengths of this study is the use of a validated WHO multi-country questionnaire that was adapted to study objectives and pretested. Another strength is that the population studied was a rural one where IPV is prevalent. A possible limitation of this study is a recall bias because the respondents may not easily recall past experiences. The figures obtained may have been under-reported because the respondents may be unwilling to volunteer certain experiences that may constitute IPV for cultural or emotional reasons.

## Conclusion and recommendation

Intimate partner violence is the most pervasive yet least recognised human rights abuse in the world. This study revealed the staggering magnitude of threats confronting human rights with the overall IPV prevalence of 79.4%. The study afforded the opportunity to estimate the size of different components of IPV with Controlling IPV ranking highest. Membership of an interest group, and the beliefs that a wife must obey her husband/partner in all things even if she disagrees and in wife’s unconditional obligation to sex were factors significantly associated with IPV.

Therefore, public awareness starting from the community heads and opinion leaders to the rest of the community should be engaged in desensitising them of the erroneous views that encourage partner violence. There should be increased advocacy for women’s rights and sexual autonomy. We also encourage women to be part of social groups that can enhance their education and empowerment as this may be a formidable tool in reducing the burden of IPV in our environment.

## References

[1] Krug EG, Dahlberg LL, Mercy JA, Zwi AB, Lozano R, editors. World report on violence and health. Geneva: World Health Organization; 2002.

[2] Onoh RC, Umeora OUJ, Ezeonu PO, Onyebuchi AK, Lawani OL, Agwu UM. Prevalence, pattern and consequences of intimate partner violence during pregnancy at Abakaliki Southeast Nigeria. Ann Med Health Sci Res. 2013;3(4):484–491. 10.4103/2141-9248.122048 24379996 PMC3868111

[3] Benebo FO, Schumann B, Vaezhasemi M. Intimate partner violence against women in Nigeria: A multilevel study investigating the effect of women’s status and community norms. BMC Womens Health. 2018;18(1):136. 10.1186/s12905-018-0628-7 30092785 PMC6085661

[4] Koenig MA, Lutalo T, Zhao F, et al. Domestic violence in rural Uganda: Evidence from a community-based study. Bull World Health Organ. 2003;81(1):53–60. 12640477 PMC2572313

[5] El-zanaty F, Hussein EM, Shawky GA, Way AA, Kisher S. Egypt demographic and health survey 1995. Calverton, MD: National Population Council and Macro International Inc.; 1996.

[6] Haj-Yahia MM, Edleson JL. Predicting the use of conflict resolution tactics among engaged Arab-Palestinian men in Israel. J Fam Violence. 1994;9:47–62. 10.1007/BF01531968

[7] Fikree FF, Bhatti LI. Domestic violence and health of Pakistani women. Int J Gynecol Obst. 1999;65(2):195–201. 10.1016/S0020-7292(99)00035-1 10405066

[8] Ezechi OC, Kalu BKE, Ezechi LO, Nwokoro CA, Ndububa VI, Okeke GCE. Prevalence and pattern of domestic violence against pregnant Nigerian women. J Obstet Gynecol. 2004;24(6):652–656. 10.1080/01443610400007901 16147605

[9] World Health Organization. WHO multi-country study on women’s health and domestic violence against women. Geneva: World Health Organization; 2005.

[10] Heise L, Ellsberg M, Gottemoeller M. Ending violence against women. Baltimore, MD: Johns Hopkins University School of Public Health; 1999.

[11] Ellsberg M, Pena R, Herrera A, Liljestrand J, Winkvist A. Candies in hell: Women’s experiences of violence in Nicaragna. Soc Sci Med. 2000;51(11):1595–610. 10.1016/S0277-9536(00)00056-3 11072881

[12] Martin SL, Kilgallen B, Tsui AO, Mantra K, Singh KK, Kupper LL. Sexual behaviours and reproductive health outcomes: Associations with wife abuse in India. JAMA. 1999;282(20):1967–1972. 10.1001/jama.282.20.1967 10580466

[13] Cunradi CB, Caetano R, Schafer J. Socioeconomic predictors of intimate partner violence among white, black, and hispanic couples in the United States. J Fam Violence. 2002;17:377–389. 10.1023/A:1020374617328

[14] Fawole OI, Aderonmu AL, Fawole AO. Intimate partner abuse: Wife beating among civil servants in Ibadan, Nigeria. Afr J Reprod Health. 2005;9(2):54–64. 10.2307/3583462 16485586

[15] Hoffman KL, Demo DH, Edwards JN. Physical wife abuse in a non-western society: An integrated theoretical approach. J Marriage Fam. 1994;56(1):131–146. 10.2307/352709

[16] Owoaje ET, OlaOlorun FM. Women at risk of physical intimate partner violence: A cross-sectional analysis of a low-income community in Southwest Nigeria. Afr J Reprod Health. 2012;16(1):43–53. 22783667

[17] Balogun MO, Fawole OI, Owoaje ET. Experience and attitude of rural women to intimate partner violence in Nigeria. J Public Health. 2013;21:333. 10.1007/s10389-013-0564-9

[18] Adeleye OA, Chiwuzie J. ‘He does his own and walks away’ perceptions about male attitudes and practices regarding safe motherhood in Ekiadolor, Southern Nigeria. Afr J Reprod Health. 2007;11(1):76–89. 10.2307/30032490 17982950

[19] Obi SN, Ozumba BC. Factors associated with domestic violence in south-east Nigeria. J Obstet Gynaecol. 2007;27(1):75–78. 10.1080/01443610601056509 17365465

[20] Ogboghodo EO, Omuemu VO. Prevalence, pattern and determinants of domestic violence among antenatal clinic attendees in a secondary health facility in Benin city, Edo State. J Community Med Prim Health Care. 2016;28(1):65–75.

[21] Oyediran KA, Isiugo-Abanihe UC. Perceptions of Nigerian women on domestic violence. Afr J Reprod Health. 2005;9(2):39–53. 10.2307/3583461 16485585

[22] Speizer IS. Intimate partner violence attitudes and experience among women and men in Uganda. J Interpers Violence. 2010;25(7):1224–1224. 10.1177/0886260509340550 19758975 PMC2877756

[23] Umana JE, Fawole OI, Adeoye IA. Prevalence and correlates of intimate partner violence towards female students of the University of Ibadan, Nigeria. BMC Womens Health. 2014;14:131. 10.1186/1472-6874-14-131 25488683 PMC4295485

[24] Abeya SG, Afework MF, Yalew AW. Health effects of intimate partner violence against women: Evidence from community based cross sectional study in Western Ethiopia. Sci Technol Arts Res J. 2013;2(2):48–57. 10.4314/star.v2i2.98884

[25] Saile R, Neuner F, Ertl V, Catani C. Prevalence and predictors of partner violence women in the aftermath of war: A survey among couples in Northern Uganda. Soc Sci Med. 2013;86:17–25. 10.1016/j.socscimed.2013.02.046 23608090

[26] Lawoyin T. Main causes of mortality and morbidity among children and women. In: Hodges A, editor. Children’s and women’s rights in Nigeria: A wake-up call. Situation assessment and analysis. Abuja: National Population Commission and UNICEF; 2001. p. 40–54.

[27] Kim J, Motsei M. ‘Women enjoy punishment’: Attitudes and experiences of gender-based violence among PHC nurses in rural South Africa. Soc Sci Med. 2002;54(8):1243–1254. 10.1016/S0277-9536(01)00093-4 11989960

[28] Jewkes R. Intimate partner violence: Causes and prevention. Lancet. 2002;359(9315):1423–1429. 10.1016/S0140-6736(02)08357-5 11978358

[29] Omuemu VO, Ogboghodo EO. Ending domestic violence against women: Assessment of knowledge and perceptions of women in Benin city, Edo state. Res J Health Sci. 2016;4(1):47–59.

[30] Uthman OA, Lawoko S, Moradi T. Factors associated with attitudes towards intimate partner violence against women: A comparative analysis of 17 sub-Saharan countries. BMC Int Health Hum Rights. 2009;9:14. 10.1186/1472-698X-9-14 19619299 PMC2718859

